# Reorganization: Its Effect upon the County Society

**Published:** 1902-02

**Authors:** 


### REORGANIZATION: ITS EFFECT UPON THE COUNTY SOCIETY.

The report of the Committee on Reorganization, read before the St. Paul meeting of the American Medical Association, suggests that in order to become a part of the American Medical Association all State and Territorial societies shall adopt a uniform organic law embodying certain fundamental principles, among which great emphasis is given to the following: First. Membership in duly recognized county or district medical societies shall constitute membership in the State society; and, second, no one shall be eligible to membership in either the State or national organizations unless he be a supporter and a member in good standing in his local society.

It is reasonably certain that most, if not all, of the State societies within a short time will have adopted, as a basis for reorganization, practically all of the recommendations contained in the committee's report, with the result that the profession throughout every State and Territory will be united in one common organization, beginning with the county and district societies and extending through the State society to the national Association.

In their efforts to perfect a scheme of reorganization, Dr. Simmons and his associates have had the wisdom to understand that no strong and useful State or national body can exist unless the county and district societies be given due recognition and be made to form a part of the general plan.

The making of membership in the county or district society an absolute prerequisite to recognition by the higher bodies will take the wind out of the sails of the fellow who now insists upon running the local society entirely his own way, or else resigns, boasting that he can hold membership in the State organization. The laity will soon learn that unless a physician is connected with his home society he has no standing in either the State or national associations, and, therefore, to suspect him of professional dishonesty. Men outside the organization will find it hard to make explanation, for it will be known that neither jealousy nor personal spite can succeed in excluding a reputable physician, since every one will have the right to appeal to the State society for instatement, or for reinstatement if he has been unlawfully expelled. Some men naturally like to flock by themselves, but hereafter such will have to sacrifice that pleasure unless they are willing to have their names associated with irregulars who cannot under any circumstances gain recognition as legitimate practitioners.

At the 1902 meeting to be held at Dallas, May 6-9, the Constitution and By-Laws of the Texas State Medical Association will undoubtedly be revised according to the plans laid down by the national committee. Then will every duly recognized county and district society throughout the State take a new lease on life, and then will the State Association become a power for good both to the public and to the profession.

